# Transient Increase in Cyclic AMP Localized to Macrophage Phagosomes

**DOI:** 10.1371/journal.pone.0013962

**Published:** 2010-11-11

**Authors:** Megan N. Ballinger, Timothy Welliver, Samuel Straight, Marc Peters-Golden, Joel A. Swanson

**Affiliations:** 1 Division of Pulmonary and Critical Care Medicine, Department of Medicine, University of Michigan Health Systems, Ann Arbor, Michigan, United States of America; 2 Department of Microbiology and Immunology, University of Michigan Health Systems, Ann Arbor, Michigan, United States of America; 3 Program in Immunology, University of Michigan Health Systems, Ann Arbor, Michigan, United States of America; University of Toronto, Canada

## Abstract

Cyclic AMP (cAMP) regulates many biological processes and cellular functions. The importance of spatially localized intracellular gradients of cAMP is increasingly appreciated. Previous work in macrophages has shown that cAMP is produced during phagocytosis and that elevated cAMP levels suppress host defense functions, including generation of proinflammatory mediators, phagocytosis and killing. However, the spatial and kinetic characteristics of cAMP generation in phagocytosing macrophages have yet to be examined. Using a Förster resonance energy transfer (FRET)-based cAMP biosensor, we measured the generation of cAMP in live macrophages. We detected no difference in bulk intracellular cAMP levels between resting cells and cells actively phagocytosing IgG-opsonized particles. However, analysis with the biosensor revealed a rapid decrease in FRET signal corresponding to a transient burst of cAMP production localized to the forming phagosome. cAMP levels returned to baseline after the particle was internalized. These studies indicate that localized increases in cAMP accompany phagosome formation and provide a framework for a more complete understanding of how cAMP regulates macrophage host defense functions.

## Introduction

Resident macrophages are essential in containing and controlling infections by recognizing and destroying invading pathogens. Phagocytosis, the process by which macrophages internalize microbes, apoptotic cells and small particles, is a highly regulated process mediated via phagocytic receptors, such as Fcγ receptors [1], pattern recognition receptors such as Toll-like receptors [2], and complement receptors [3]. Once internalized, microbes are confined to an organelle known as the phagosome, which allows them to be targeted for killing by a variety of microbicidal mechanisms while remaining segregated from the rest of the cell [4].

One important regulator of macrophage function is the second messenger, cyclic adenosine monophosphate (cAMP) [5]. The generation of cAMP is initiated when a ligand binds to a G protein-coupled receptor, stimulating the enzyme adenylyl cyclase (AC) to catalyze the cyclization of ATP. The production of cAMP within the cell is tightly regulated, in part through activities of cytoplasmic phosphodiesterases (PDEs) [6]. The intracellular signaling of cAMP is coordinated primarily through two effector molecules: protein kinase A (PKA) and exchange proteins directly activated by cAMP (Epac) [7]. Previous work has shown that PKA and Epac can have distinct, redundant, or even opposing effects within the same cell, and both play important roles in modulating host defense functions in macrophages [8], [9].

cAMP serves as a negative regulator of phagocyte function [10] and elevated cAMP levels are associated with suppression of innate immune functions including the production of pro-inflammatory mediators, phagocytosis, and microbial killing [5]. Early biochemical and fixed cell microscopy studies indicated that intracellular cAMP production in macrophages and neutrophils increases during phagocytosis [11], [12], [13], [14], through regulation by PDEs [15]. More recent work has shown that PDEs play an important role in creating discrete subcellular pools of cAMP within the cell, with higher levels of cAMP found at the plasma membrane and within the nucleus and lower levels in the cytosol [16].

Studies employing classical biochemical and fixed-cell microscopy approaches [17] obtain suboptimal kinetic and spatial resolution of cAMP pools. In recent years, the use of techniques based on Förster resonance energy transfer (FRET) have allowed monitoring of cAMP levels in live cells [18]. This provides better spatial and kinetic information about intracellular cAMP dynamics [19]. FRET microscopy has demonstrated that cAMP compartmentalization plays an important role in mediating intracellular signaling events [20], [21], [22]. A wide range of cAMP biosensors have been utilized, and these differ considerably in their localization, dynamic range, temporal resolution and signal-to-noise ratios [18].

Other quantitative fluorescence microscopic studies have allowed the component activities of phagocytosis to be resolved into distinct and characteristic patterns [23], [24], [25]. Some activities are restricted to the early processes, such as extension of the phagocytic cup, while other activities correspond to later processes such as cup closure. To improve the temporal resolution of cAMP signaling during phagocytosis and to put cAMP signaling in the context of other signaling activities, this study used an Epac-based biosensor and FRET microscopy to measure localized changes in this second messenger during Fcγ receptor-mediated phagocytosis by macrophages. Although no differences in total cellular cAMP levels were detectable, either biochemically or by FRET live-cell microscopy, a transient burst of cAMP production was demonstrable in the immediate vicinity of the forming phagosome.

## Materials and Methods

### Cell culture and transfection

RAW264.7 macrophage-like cells (American Type Culture Collection) were cultured as previously described [24]. To prepare for microscopy, cells were plated at ∼5×10^5^ cells per coverslip (25 mm circular, No. 1.5) and transfected with plasmids using Roche FuGENE 6 according to the manufacturer's protocol (Roche Diagnostics). Coverslips were assembled into temperature-controlled chambers and cells were cultured in Ringers buffer [24]. To measure phagocytosis, opsonized sheep erythrocytes were prepared and added to the macrophages as previously described [26].

### Plasmids and protein purification

The Epac1-camps plasmid [27] (generously provided by Martin Lohse, University of Wurzburg) was either used directly or its YFP domain was mutated to Citrine (YFP-Q69M) by the Quickchange Method (Stratagene). In addition, plasmids for monomeric CFP (mCFP), monomeric citrine (mCit), linked mCFP-YFP (G4) and linked mCFP-mCit (C4) were also used as experimental and ratiometric controls [28]. All DNA sequences were confirmed at the University of Michigan DNA Sequencing Core.

### cAMP biochemical assay

RAW264.7 cells were cultured overnight and incubated with the AC activator forskolin (200 µM) or the *Bacillus anthracis* Edema Toxin (EdTx) (1.0 µg/ml protective antigen and 0.5 µg/ml edema factor; BEI Resources, Manassas, VA), a microbial AC [29], for the indicated time intervals. During the last 20 min of the incubation, opsonized sRBCs were added at a ratio of 20∶1. After treatments, cells were lysed with 0.1 M HCl and intracellular cAMP levels were determined by ELISA according to manufacturer's instructions (Cayman Chemical) [30].

### Image acquisition and processing

Images were collected using a wide field Nikon Eclipse TE-300 inverted microscope as previously described [31]. Excitation and emission filters were positioned to visualize mCFP (I_D_): excitation (ex) 430±12.5 nm, emission (em) 470±15 nm; mCit or YFP (I_A_): ex 500±10 nm, em 535±15 nm; and FRET (I_F_): ex 430±12.5, em 535±15 nm. A series of four images (phase-contrast, I_D_, I_A_, and I_F_) were recorded every 30 seconds. The FRET calculator (Center for Live Cell Imaging, University of Michigan, available on request) was used to perform image processing based on the equations of FRET stoichiometry [28]. Briefly, I_D_, I_A_, and I_F_ images were corrected first by subtracting camera noise (bias correction) and then for unevenness in the field of illumination (shading correction). FRET calibration constants were obtained for the microscope using images of cells expressing mCit, mCFP and the control linked mCFP-mCit molecule (C4 control). The calibrated microscope was then used to calculate a corrected fluorescence FRET image (E_A_), which reports FRET efficiency corrected for variations in cell thickness. To measure relative changes in donor and acceptor photobleaching. the molar ratio of mCit to CFP (R_I_) was calculated for each image.

### Particle tracking and processing

The particle tracking algorithm in the FRET calculator was used to measure differences in E_A_ and R_I_ at and around the forming phagosome (regions R1-R5), and in the total cell (T_C_), in cells expressing either Epac-camps or C4 control. A 2.3 µm radius circular region (R1) was drawn around the target erythrocyte and 4 additional concentric regions with radii of 4.6 µm (R2), 6.9 µm (R3), 9.2 µm (R4) and 11.5 µm (R5) were drawn. The target was tracked on the phase-contrast image throughout the time series and was used to position the measurement circles. To synchronize multiple phagocytic events, the beginning of phagocytosis was identified in each time series as the first frame in which pseudopod extension and cup formation were detectable (as seen in the I_A_ image). To account for differential photobleaching of donor and acceptor, E_A_ for each concentric region (R1-R5) was divided by E_A_ for the entire cell (T_C_). To determine differences between background FRET and intracellular cAMP production, the normalized E_A_ Epac-camps R1-R5 was subtracted from the corresponding normalized E_A_ C4 R1-R5 images. To verify that observed changes were not due to acceptor photobleaching, the same analysis was performed using the R_I_ images from C4 control- and Epac-camps-expressing cells.

### Statistical analysis

Data are presented as mean ± SEM and were analyzed with the Prism 4.0 statistical program (GraphPad Software). The group means for different treatments were compared by ANOVA. When significant differences were identified, individual comparisons were subsequently analyzed using an unpaired *t* test with Bonferroni correction. Statistical significance was set at a *p* value <0.05.

## Results

### Biochemical and FRET-based cAMP measurements in macrophages

**Figure 1:**
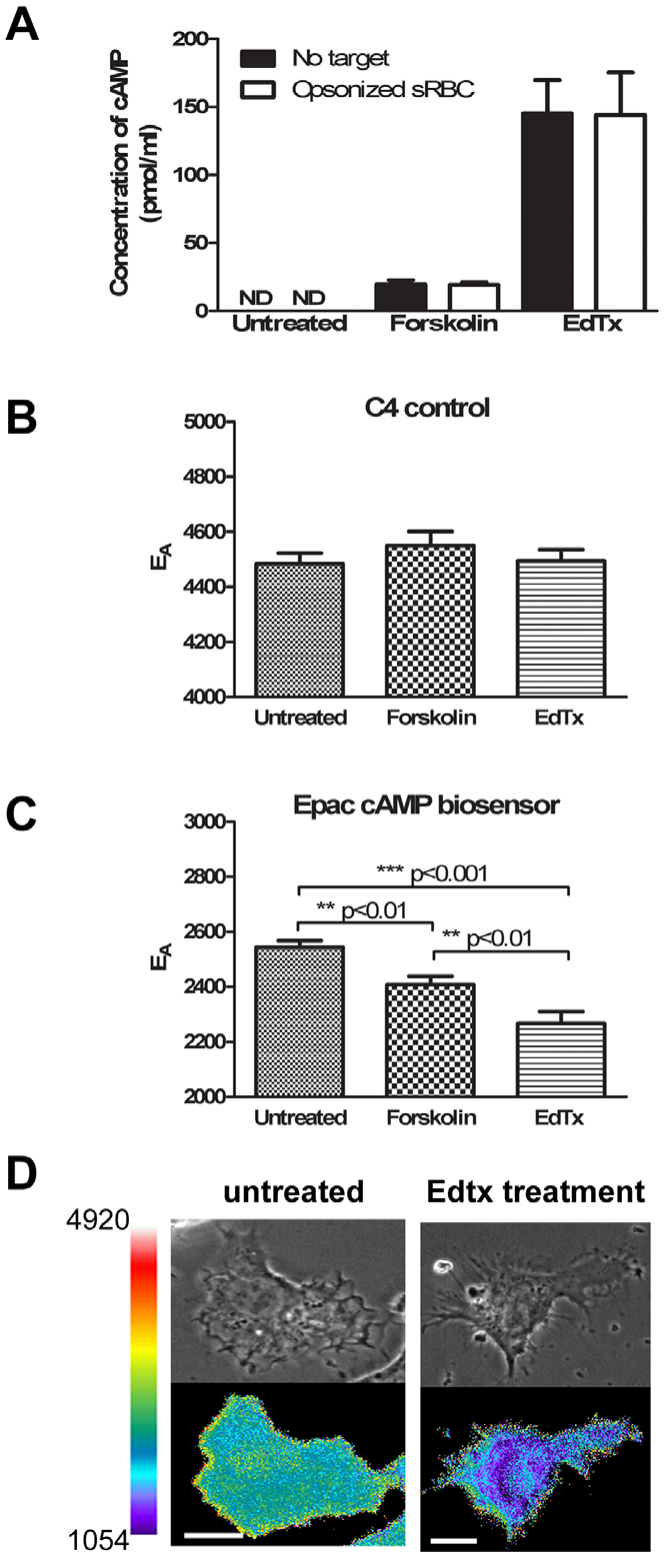
Changes in cAMP measured by biochemical and FRET microscopic methods in macrophages. A. RAW cells were plated overnight at 2×10^6^ cells per well and stimulated with 200 µM forskolin for 20 min or with EdTx for 3 h. During the final 20 min incubation, either PBS (blank bars) or opsonized sRBCs (open bars) were added to cells as indicated. Total cAMP was quantified as described in material and methods. Data represent the mean ± SEM. B. and C. Cells expressing C4 control (B) or the Epac-camps biosensor (C) were either analyzed directly or following treatment with the indicated compounds. The relative amount of FRET after each condition was determined and the results are graphed as mean ± SEM (n = 50–100 cells per condition). D. A representative phase-contrast (top) and corresponding E_A_ image (bottom) of an untreated or EdTx-treated macrophage. Color bar indicates scale of ratio and scale bar is 10 µm.

To verify that changes in cAMP levels could be detected using these methods, RAW cells were incubated with forskolin for 20 min or EdTx for 3 h (based on pilot studies showing that these incubation times yielded maximal increases in cAMP) in the presence or absence of an opsonized phagocytic target (Figure 1A). cAMP was not detectable by ELISA in untreated RAW cells, but increased to measurable levels following addition of either forskolin or EdTx. EdTx resulted in 7.5-fold greater levels of cAMP than did forskolin. The addition of a phagocytic target had no effect on total cAMP levels measured in macrophages otherwise incubated with forskolin or EdTx alone (Figure 1A).

To localize cAMP in live macrophages, we used a previously described biosensor, Epac-camps, which distributes uniformly through cytoplasm in cells [27]. Binding of cAMP causes a conformational change in Epac-camps, resulting in a decreased FRET signal [27]. Preliminary studies optimized the Epac-camps biosensor for measuring cAMP in macrophages during phagocytosis. Preliminary experiments using the linked mCFP-YFP (G4) revealed cAMP-independent changes in FRET during phagocytosis. We reasoned that these changes in FRET signal resulted from decreases in cytoplasmic pH near forming phagosomes, as YFP fluorescence can be affected by cytoplasmic pH [23]. We therefore improved the mCFP-YFP Epac-camps by creating a single point mutation in its YFP domain (Q69M), creating a mCFP-mCIT Epac-camps biosensor that was relatively unaffected by fluctuations in cytoplasmic pH [28].

RAW cells were transfected with either the C4 control plasmid (mCFP covalently linked to mCit) or the mCFP-mCit Epac-camps biosensor and FRET was detected as the processed E_A_ image. E_A_ in cells transfected with the C4 control plasmid was 0.448±0.004 (Figure 1B). Likewise, cells transfected with the modified Epac-camps biosensor had a measured E_A_ value of 0.254±0.002 (Figure 1C). When cells were incubated with forskolin, E_A_ decreased 5% in Epac-camps-expressing cells, indicating an increase in intracellular cAMP. E_A_ of control C4-expressing cells was unchanged after incubation with forskolin. Furthermore, EdTx treatment produced a 10% decrease in E_A_ in Epac-camps-expressing cells, but no change in the control cells. Thus, the biosensor-derived measurements confirmed the biochemical data indicating that EdTx was a more potent enhancer of intracellular cAMP than was forskolin. Attempts to measure decreases in intracellular cAMP elicited by incubating cells with an adenylyl cyclase inhibitor (SQ-22536) and a Gαi-coupled ligand, leukotriene B_4_. did not detect any increases in FRET signal under either of these conditions This indicated that resting cellular cAMP levels are on the low end of the probe's dynamic range, such that most probe molecules in cytoplasm do not contain bound cAMP. Thus, a decrease in cAMP concentrations would not be reported by the Epac-camps biosensor. Our data thus confirm that RAW cells can make cAMP in response to stimulation and cAMP increases at the whole cell level can be measured both biochemically and by using a FRET-based cAMP biosensor.

### Effects of macrophage phagocytosis on total cellular cAMP levels

**Figure 2:**
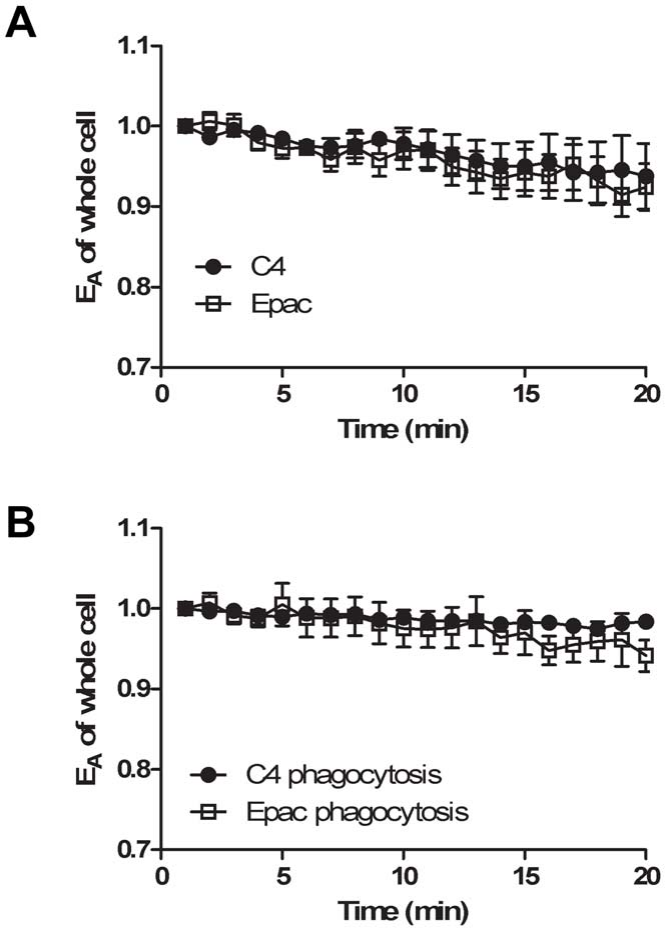
No change in total cAMP during phagocytosis. RAW cells were transfected with plasmids for C4 control or the Epac-camps biosensor. Total cellular E_A_ was measured over time and plotted relative to the first measured value. A. Measurements of unfed macrophages showed small decreases in FRET, indicating selective photobleaching of mCit. B. Transfected cells were fed opsonized targets and phagocytosis was synchronized as described in material and methods. No significant changes in cAMP were detectable during phagocytosis. Results are shown as mean ± SEM of 4–7 cells.

Having verified that changes in intracellular cAMP could be measured in live cells using the modified Epac-camps biosensor, we next examined cAMP levels in macrophages during phagocytosis. RAW cells were transfected with either the control C4 plasmid or the plasmid encoding the Epac-camps biosensor, and the total cellular FRET was measured in individual cells during 20 min incubations with or without opsonized sRBCs (Figure 2). Intracellular expression levels of the C4 control and the Epac-camps biosensor were similar to each other. Additionally, all transfected cells bound and internalized opsonized targets at rates similar to non-transfected cells (internalization completed within approximately 7 min). During imaging of live cells, the total cell E_A_ decreased slightly over time (Figure 2A) in both the Epac-camps and the C4 control cells, indicating acceptor photobleaching. This was supported by measurements of R_I_, which also indicated minor photobleaching. However, the rates of decrease in E_A_ were the same for C4 control and the Epac-camps. Addition of opsonized targets did not produce significant changes in total E_A_ values or the rates of E_A_ decrease due to photobleaching (Figure 2B).

**Figure 3:**
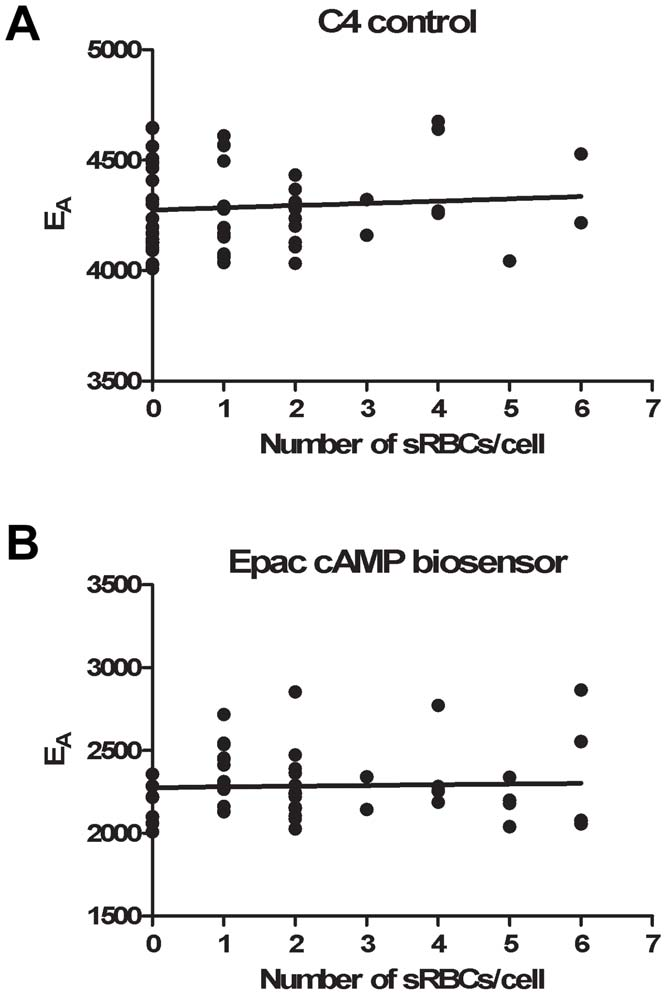
Levels of intracellular cAMP are independent of the number of particles phagocytosed. RAW cells were plated at 5×10^5^ cells per coverslip and transfected with plasmids encoding the C4 control (A) or the mCFP-mCit Epac-camps biosensor (B). Opsonized sRBCs were then added to the cultures and cells were permitted to phagocytose for 1 h at 37°C. Non-ingested sRBCs were washed away and the images were collected and analyzed. There was no significant correlation between E_A_ and the number of sRBCs ingested in the C4 control (A, p = 0.5509) or Epac-camps (B, p = 0.7879) cells, when analyzed by linear regression (n = 50–52 cells per condition).

To investigate whether the number of particles ingested correlated with changes in intracellular cAMP concentrations, macrophages were incubated with opsonized sRBCs for 1 h and then cAMP was measured using FRET microscopy (Figure 3). Calculation of E_A_ as a function of the number of ingested particles revealed that total cellular cAMP levels were constant at all phagocytic loads (Figures 3A and B).

### Levels of cAMP near forming phagosomes

**Figure 4:**
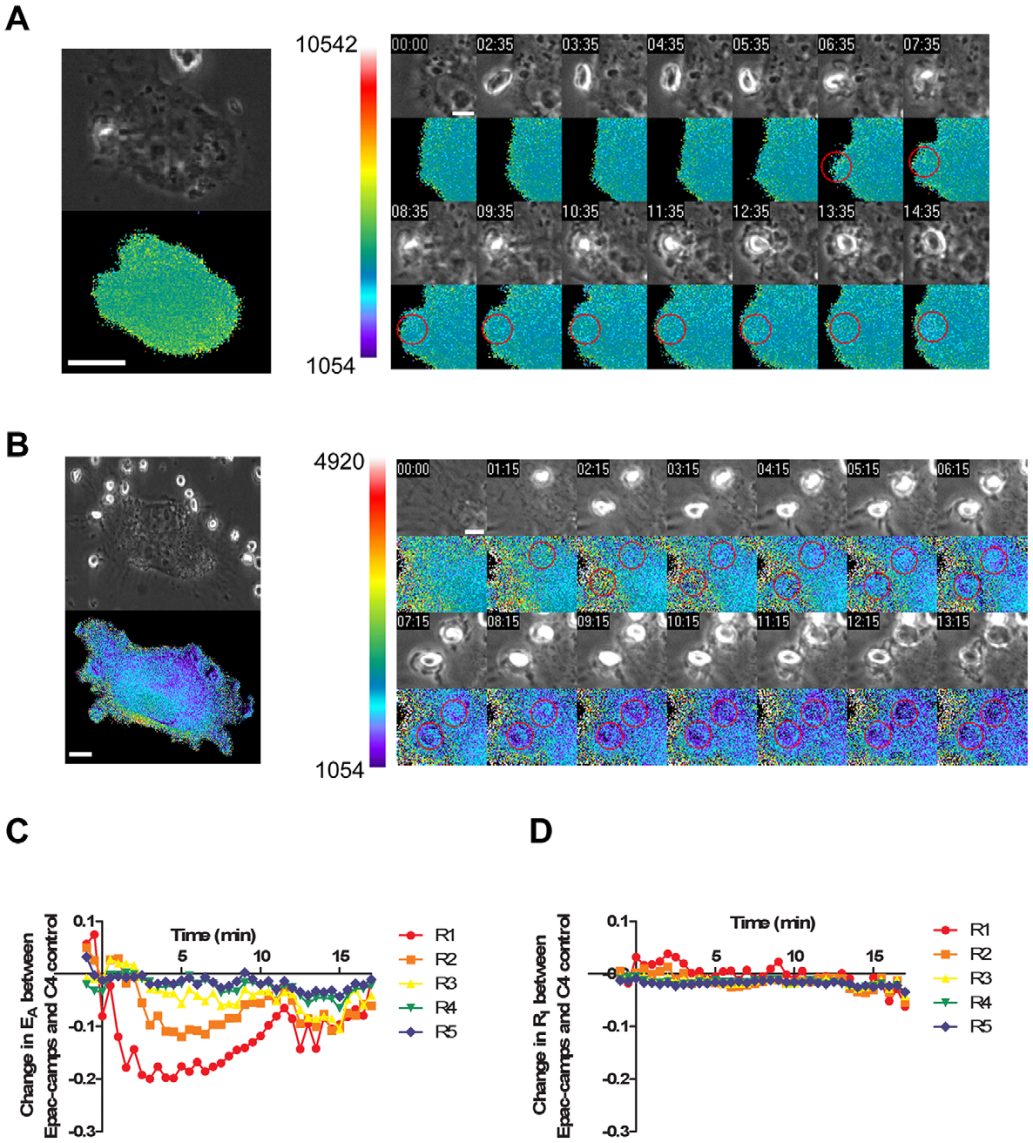
Transient burst of cAMP at the developing phagosome. RAW cells expressing C4 or the Epac-camps biosensor were fed opsonized targets and component images for phase-contrast and FRET were taken every 30 sec to capture the phagocytic process from initiation to closure of the phagocytic cup. A. and B. Left insert: A phase-contrast (top) and corresponding E_A_ image (bottom) of intact live macrophages transfected with C4 control (A) or Epac-camps (B), from the designated time intervals. Right inserts: One minute time course of a magnified portion of the cell transfected with the specified plasmid (beginning immediately after the sRBCs were added to the culture). The red circle denotes the location of the opsonized sRBC on the E_A_ image. Color bar indicates scale of ratio and scale bar is 10 µm in the left insert and 5 µm.in the right insert C. To normalize for non-specific cAMP-mediated effects during phagocytosis, the data are shown as the phagosome-specific difference in E_A_ between C4 control and Epac-camps (ec) biosensor-expressing cells. (ie., (E_A(C4-phago)_/E_A(C4-cell)_) − (E_A(ec-phago)_/E_A(ec-cell)_); n = 10 cells) D. To verify that the differences seen between cells transfected with the C4 control construct and the Epac-camps construct were not due to selective bleaching of one of the fluorescent proteins, the R_I_ values are plotted (ie., (R_I(C4-phago)_/R_I(C4-cell)_) − (R_I(ec-phago)_/R_I(ec-cell)_)).

We next examined subregions of cells to determine whether cAMP levels increased near the forming phagosomes. Cell expressing either C4 control or Epac-camps were incubated with opsonized sRBCs and the amount of total cellular FRET as well as the FRET in concentric regions of interest around the forming phagosome were measured. The results were plotted as the difference between signals from Epac-camps and C4 control cells in each of the subregions, processed for both E_A_ (Figure 4A) and R_I_ (Figure 4B). The interaction between the opsonized target and cellular Fcγ receptors resulted in a transient gradient of cAMP radiating from the forming phagosome. This was indicated by significant changes in E_A_ in Epac-camps-expressing cells. Regions closest to the phagosome (R1 and R2) exhibited significantly elevated cAMP (Figure 4A and D). Levels of cAMP increased at the plasma membrane approximately 1 min after the initiation of phagocytosis, remained high for several minutes, and then returned to baseline levels following completion of phagosome formation. We observed no changes in E_A_ in cells expressing C4 control (Figure 4C), confirming that the changes observed in Epac-camps-expressing cells were indeed due to changes in intracellular cAMP. In addition, the mCit/mCFP ratio R_I_ did not change significantly during phagocytosis by Epac-camps-expressing cells (Figure 4B), further indicating that the changes in E_A_ did not reflect selective photobleaching of fluorescent proteins.

## Discussion

This study investigated the spatial and temporal dynamics of cAMP in live phagocytosing macrophages. Using cAMP FRET biosensors, we show that levels of cAMP rise quickly at the nascent phagocytic cup and return to baseline following internalization of the particle. The timing of this localized rise in cAMP indicates that it contributes to phagosome formation.

Previous studies measuring cAMP in phagocytes with biochemical assays and fixed-cell microscopy were limited in their temporal resolution. Using radioimmunoassays, total cAMP was measured at different time points during Fcγ receptor-mediated phagocytosis in neutrophils or Kupffer cells. Increases in total cAMP were observed between 30 sec and 15 min after addition of an opsonized target and those levels returned to baseline after 3 and 60 min [11], [12], [13]. Studies of fixed neutrophils using an antibody against cAMP showed a uniform distribution of cAMP throughout the cytoplasm of unstimulated cells [14]. Upon phagocytic challenge with an opsonized target, higher concentrations of cAMP were localized to the forming phagosome [14].

The FRET microscopic method introduced here has the advantages of providing high specificity for cAMP and good temporal and spatial resolution. Its disadvantages include a weak signal and a small dynamic range. Mutation of YFP to Citrine in Epac-camps improved the probe's specificity for cAMP by reducing potential artifacts resulting from local fluctuations in cytoplasmic pH. The probe was bright enough to permit image acquisition every 30 seconds, allowing measurement of localized increases in cAMP throughout the 7- to 8-minute process of phagocytosis. However, like most linked FRET biosensors, the Epac-camps probe exhibited a limited dynamic range. Binding of cAMP to Epac-camps resulted in a small decrease in FRET efficiency: the fluorescent proteins are bright but the measurable shift in FRET is a weak signal. Such small differences in FRET efficiency can be problematic when trying to detect localized signals surrounded by cytoplasm, especially in thick cells. Moreover, the maximum and minimum FRET efficiencies reported by the biosensor are restricted to the small range of cAMP concentrations above and below the binding affinity of Epac. The weak signal and small dynamic range of Epac-camps explain why the decrease in FRET signals from cells treated with EdTx or forskolin were less dramatic than the increases in signals reported by the biochemical assay. Likewise, the failure of the probe to report decreases in cAMP concentrations indicates that concentrations of cAMP in unstimulated macrophages are at or below the lower limit of detection by Epac-camps. Thus, the probes were adequate to report transient increases in cAMP near forming phagosomes but would have likely missed smaller foci of elevated cAMP or any local decrease in cAMP. Although we did not observe any differences in bulk cAMP during macrophage phagocytosis by RAW macrophages (Figure 2), a transient increase in cAMP was localized to the forming phagosome (Figure 4).

Previous work has shown that elevated cAMP inhibits both internalization of opsonized particles [32] and the recruitment of proteins necessary for pathogen destruction [9]. Although only the phagocytosis of opsonized sRBCs was utilized in this assay, we hypothesize that there would also be different levels of cAMP generated in response to other internalized particles, particularity bacteria. Previous work has shown using fixed-cell microscopy and biochemical assays that cAMP is transiently localized to the forming phagosome when the cells are ingesting opsonized zymosan particles [11], [12], [13], [14], [15]. Additionally, increased levels of cAMP have been linked to reduced actin assembly, inhibition of phagosome-lysosome fusion and acidification, and increased intraphagosomal growth of pathogens [33]. For these reasons, our results showing transient elevation of cAMP during the initial formation of the phagosome appear surprising. It is possible that a transient and localized burst of cAMP plays a role in mediating phagosome formation, although further studies will be necessary to clarify the functional importance of this finding. Additional work is also needed to further elucidate the spatial and kinetic effects of cAMP on phagosome trafficking and eventual pathogen destruction.

The regulation of intracellular cAMP is essential to a variety of signal transduction events within cells. Previous work has shown a correlation between the amount of cAMP produced at the phagosome and the ability of the cell to internalize and kill an invading pathogen [33]. The importance of cAMP as a negative regulator of phagocyte function is further indicated by the fact that several pathogenic microorganisms elevate cAMP in target host cells [5]. Pathogens use cAMP to disable phagocytosis, intracellular killing and inflammatory mediator generation, thus allowing the pathogen to gain an advantage against the host. Perhaps premature elevation of cAMP by toxins or immunomodulatory compounds inhibits phagocytosis by prematurely inactivating essential early activities.

These studies extend our understanding of cAMP signaling in phagocytosing macrophages by putting its dynamics into the context of signaling for phagocytosis. This is essential for understanding host pathogen interactions and the immunomodulatory effects of therapeutic agents that modulate cAMP levels.
